# Does the presence of unilateral gingival recession on maxillary canines influence smile esthetics?

**DOI:** 10.1590/2177-6709.25.1.056-063.oar

**Published:** 2020

**Authors:** Bruna Alecrim Figueiredo, Joanna Betrine Pereira Ribeiro, Andre Wilson Machado

**Affiliations:** 1 Universidade Federal da Bahia, Faculdade de Odontologia (Salvador/BA, Brazil).; 2 Universidade Federal da Bahia, Faculdade de Odontologia, Departamento de Ortodontia (Salvador/BA, Brazil).

**Keywords:** Orthodontics, Dental esthetics, Smile

## Abstract

**Objective:**

The objective of this study was to determine orthodontists’, periodontists’, and laypersons’ perception of smile esthetics, regarding the presence of different levels of gingival recession on the maxillary left canine.

**Material and Methods:**

Two close-up smile images (frontal and oblique) of a white female were selected for this study. The images were digitally altered to create different levels of gingival recession on maxillary left canine, in 0.5-mm increments. They were randomly arranged into a photo album that was shown to 135 evaluators: 45 orthodontists, 45 periodontists, and 45 laypersons. Each evaluator was asked to rate the smile attractiveness, using to a visual analog scale. Data were analyzed statistically using ANOVA, Tukey’s *post-hoc* test, and Student t-test.

**Results:**

According to the orthodontists and periodontists, all levels of recession were considered as unesthetic in both types of images. According to the laypersons, gingival recession > 1.5 mm in the frontal image and > 1.0 mm in the oblique image were considered unesthetic.

**Conclusion:**

The results showed that the presence of unilateral gingival recession on maxillary canines may negatively influence smile attractiveness, depending on the evaluator type and the level of the recession.

## RESUMO

**Objetivo:**

o objetivo do presente estudo foi determinar a percepção estética do sorriso, por ortodontistas, periodontistas e leigos, em relação à presença de recessão gengival no canino superior esquerdo.

**Métodos:**

foram selecionadas duas imagens aproximadas do sorriso (frontal e oblíqua) de uma mulher leucoderma. As imagens foram manipuladas para criar diferentes níveis de recessão gengival no canino superior esquerdo, em incrementos de 0,5 mm. Foram, então, distribuídas aleatoriamente em um álbum, que foi entregue a 135 avaliadores: 45 ortodontistas, 45 periodontistas e 45 leigos. Cada avaliador foi convidado a avaliar a atratividade das imagens em uma escala visual analógica. Os dados foram analisados estatisticamente pela ANOVA e pós-teste de Tukey e teste *t* de Student.

**Resultados:**

para ortodontistas e periodontistas, todos os níveis de recessão foram detectados como antiestético, em ambas as formas de visualização. Para os leigos, na vista frontal, a recessão gengival foi considerada antiestética a partir de 1,5 mm; enquanto na vista oblíqua, a partir de 1,0 mm.

**Conclusão:**

os resultados demonstraram que a presença de recessão gengival em caninos superiores pode impactar negativamente a atratividade do sorriso, dependendo do tipo de avaliador e da magnitude da recessão.

## INTRODUCTION

In recent years, the search for facial esthetic treatments by patients from all around the world presented a significant growth.^1^ Some studies have shown that the improvement in dental esthetics promotes a significant increase in the quality of life, emphasizing the psychosocial importance of an attractive smile.^2^ ^,^ ^3^ In Dentistry, this is evidenced in several specialties focussed in improving smile esthetics, such as Periodontics, Orthodontics, and Prosthodontics, among others.

The study of smile esthetics is complex, due to the difficulty in standardizing a realistic model and changing the variables of interest^4^. Accordingly, in order to establish more objective criteria about smile esthetics, several studies have used digital image manipulation to determine the perception of smile esthetics according to some variables such as the smile arch,^5^ ^,^ ^6^ amount of gingival display,^7^ ^-^ ^9^ different types of buccal corridor,^8^ ^,^ ^10^ ^,^ ^11^ presence of dental and gingival asymmetries in the esthetic zone^7^ ^,^ ^8^ ^,^ ^12^ ^-^ ^15^, influence of midline diastemas,^6^ ^,^ ^16^ ^,^ ^17^ impact of midline deviations and changes in the long axes of the central incisors,^7^ ^,^ ^8^ ^,^ ^12^ and the role of symmetry and proportion of maxillary central incisors.^13^ ^,^ ^18^ ^-^ ^20^

It is important to remember that esthetic perception varies between individuals.^3^ For example, the literature suggests that orthodontists are more rigorous than laypersons in detecting small deviations in smile.^6^ ^,^ ^7^ ^,^ ^11^ ^,^ ^12^ ^,^ ^19^ ^,^ ^20^ Therefore, some deviations are not often noticeable to laypersons, which may question the real need for esthetic treatments. Among several situations that may influence smile esthetics, gingival recession needs to be carefully evaluated, because of its frequent occurrence.^21^ With regard to gingival recession on canines, although the literature describes some treatments for this problem,^22^ ^-^ ^24^ only one study was found on the influence of these recessions on the perception of smile esthetics.^14^ The authors found that a 2-mm unilateral canine gingival recession was rated as unattractive.^14^ Although this information is clinically important, the question whether recessions smaller than 2 mm may impact on smile esthetics still remains undisclosed. Finding an answer in this matter is extremely important because if small recessions cannot be detected, from an esthetic standpoint, it might be unnecessary to treat them.

After analyzing this information, other questions arose, such as: what level of unilateral gingival recession on upper canines is considered unesthetic? In other words, it is important to establish the level of gingival recession that is considered acceptable, in order to help clinicians in the choice of recommending therapeutic procedures.

Therefore, the aim of this study was to determine orthodontists’, periodontists’, and laypersons’ perception of smile esthetics, regarding the presence of different levels of gingival recession on the maxillary left canine, in frontal and oblique smile views.

## MATERIAL AND METHODS

This study was approved by the Research Ethics Committee of the Federal University of Bahia, protocol no. 1.023.061, and registered by CONEP: 40677014.6.0000.5024. All participants signed an informed consent form.

Pilot study data were used in the sample size calculation using the Biostat software (version 5.0, Instituto Mamirauá, Tefé, Amazonas, Brazil). Based on the level of significance (alpha) of 0.01 and the effect size of 0.90, the sample size was calculated, based on the difference of the means, to achieve 80% power: it was shown that a minimum of 45 subjects was required for each group of evaluators.^6^ ^,^ ^9^ ^,^ ^11^ ^,^ ^13^ ^,^ ^14^ ^,^ ^19^ ^,^ ^20^

Two standardized photographs of the close-up smile (frontal and oblique) of a 30-year-old white female with a pleasant smile were selected. The woman presented healthy maxillary incisors with no restorations, and a 1.5-mm gingival recession on the left canine. The smiles were considered attractive according to some ideal parameters previously published,^6^ ^,^ ^9^ ^,^ ^11^ ^,^ ^13^ ^,^ ^19^ ^,^ ^20^ ^,^ ^25^ ^,^ ^26^ and had the following characteristics: maxillary central incisors symmetry and no upper anterior diastema,^6^ ^,^ ^8^ ^,^ ^16^ ^,^ ^19^ ^,^ ^20^ ^,^ ^25^ adequate proportion between the teeth in the esthetic zone, proper smile arch design,^6^ ^,^ ^19^ ^,^ ^20^ ^,^ ^25^ ^,^ ^26^ and gingival display of less than 1.0 mm at the maxillary central incisor region^6^ ^,^ ^7^ ^,^ ^9^ ^,^ ^17^ and of 3.0 mm at the posterior region.

The selected photographs were digitally altered using Adobe Photoshop (CS3, Adobe Systems, San Jose, California) to create symmetric images. In both photographs, the original gingival recession of the left canine was removed, correcting the gingival margin asymmetry. The image was then condensed to achieve an image with measurements identical to those on the actual patient. Thus, each millimeter measured on the digital and printed image was equivalent to each millimeter measured clinically on the patient, using the height of the maxillary right central incisor as a reference for the frontal view, and the height of the maxillary right canine for the oblique view.^6^ ^,^ ^11^ ^,^ ^13^ ^,^ ^19^ ^,^ ^20^ ^,^ ^27^

**Figure 1:**
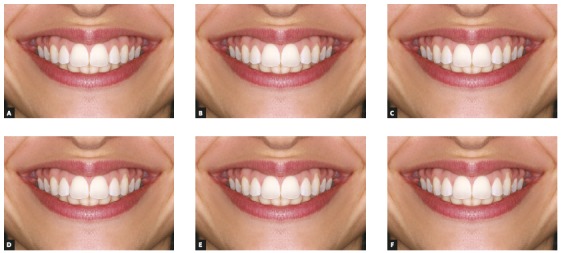
Frontal view of all unilateral gingival recession smiles, in 0.5-mm increments: A) 0 mm; B) 0.5 mm; C) 1.0 mm; D) 1.5 mm; E) 2.0 mm; F) 2.5 mm.

**Figure 2:**
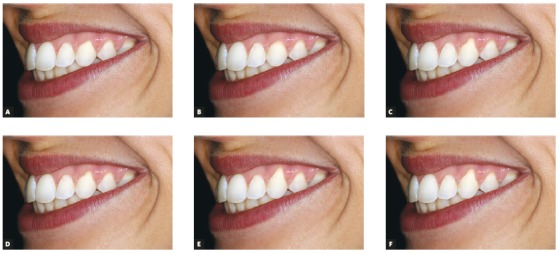
Oblique view of all unilateral gingival recession smiles, in 0.5-mm increments: A) 0 mm; B) 0.5 mm; C) 1.0 mm; D) 1.5 mm; E) 2.0 mm; F) 2.5 mm.

After the above procedures, the control smile (symmetrical, with no gingival recession) was manipulated to create 12 new smiles: 6 in frontal view (Fig 1) and 6 in oblique view (Fig 2). In order to do this, the gingival margins were apically displaced, leading to an increase in the tooth length and root surface exposure, using the patients’ original gingival recession lesion as a template to create all other smiles. Each new smile was altered in 0.5-mm increments in the cervical region of the left canine, to obtain different levels of gingival recession (0 mm, 0.5 mm, 1 mm, 1.5 mm, 2 mm, and 2.5 mm).

Final images had a resolution of 300 dpi and were professionally printed with specialized digital equipment on A4 size (21.0 x 29.7 cm)photographic papers.^6^ ^,^ ^11^ ^,^ ^13^ ^,^ ^19^ ^,^ ^20^ A photographic album was then compiled with all 12 photographs, which were randomly arranged and coded using numbers and letters.

The album was evaluated by 135 evaluators, 45 orthodontists, 45 periodontists, and 45 laypersons with college degree other than Dentistry. Before each evaluation, the panel members were informed the following information about the study: *This study is about smile esthetics perception, in which you will be evaluating a series of smile images in a photographic album, within a maximum time of 25 minutes to complete the survey, and your opinion will not be exposed to the public*. Each observer rated the attractiveness of the photographs on a form containing 12 visual analogue scales (one for each image), as reported in previous studies.^6^ ^,^ ^7^ ^,^ ^11^ ^,^ ^12^ ^,^ ^13^ ^,^ ^17^ ^,^ ^19^ ^,^ ^20^ ^,^ ^27^ The scales were anchored by word descriptors at each end: *“unattractive”* at the left, and *“very attractive”* at the right. A center mark was made on the scale to help the panel members visualize the average level of smile attractiveness. The scores were measured in millimeters by the first author, using an electronic digital caliper (Starrett, Suzhou, China).

To test the reliability of the method, 36 examiners (12 orthodontists, 12 periodontists and 12 laypeople) were randomly selected to evaluate 2 pages of photographs (1 containing all 6 images in the frontal view, and another containing 6 images in the oblique view) with 2 identical images on the same page^.6,11,13,19,20,27^ Then the scores corresponding to the 2 identical images were tabulated and examined by the intraclass correlation. High levels of reliability were found: 0.83 for orthodontists, 0.90 for periodontists, and 0.69 for laypersons.

Statistical analysis was carried out using the SPSS software (v. 16.0, SPSS Inc., Chicago, IL, USA). Descriptive statistics was used to describe the distribution of data, and means and standard deviations were calculated. 

## RESULTS

From the orthodontist’s standpoint, the most attractive smile in the frontal view was the one with no gingival recession, and the least attractive were those with gingival recession of 2.0 and 2.5 mm. Similarly, periodontists found that the most attractive smile was the one with no gingival recession, and the least attractive were those with 1.5, 2.0 and 2.5 mm of recession. Laypersons indicated as the most attractive smiles those with no gingival recession, 0.5 mm and 1.0 mm of recession. They also found that the least attractive smiles were those with 1.5, 2.0, and 2.5 mm of gingival recession (Table 1, ANOVA and Tukey’s *post-hoc* test).

**Table 1:** Means and standard deviations of smile attractiveness scores of the frontal view, according to the three groups of evaluators.

Gingival Recessions	Orthodontists (O)			Periodontists (P)			Laypersons (L)			O x P x L
	Mean	SD	Result*	Mean	SD	Result*	Mean	SD	Result*	**
0.0 mm	73.81	26.18	A	82.96	20.40	A	78.88	17.59	A	O = P = L
0.5 mm	59.73	25.94	B	62.84	19.02	B	70	17.75	A,B	O = P = L
1.0 mm	52.01	22.81	B,C	53.86	21.23	B	68.93	18.80	A,B	(O = P) < L
1.5 mm	40.80	19.71	C	39.74	18.36	C	56.48	20.42	B,C	(O = P) < L
2.0 mm	32.81	21.85	D	32.19	20.16	C	51.48	24.57	C	(O = P) < L
2.5 mm	34.61	20.51	D	31.92	21.19	C	49.23	25.4	C	(O = P) < L

* Letters arranged in order according to smile attractiveness (ANOVA and Tukey’s post-hoc test): same capital letters in the column do not differ statistically.

**Comparison among orthodontists, periodontists, and laypersons (ANOVA and Tukey’s post-hoc test).

Similar results were found in the oblique view. For the group of orthodontists, the most attractive smile was the one with no gingival recession, and the least attractive were those with gingival recession of 1.5, 2.0, and 2.5 mm. Following the same trend, periodontists indicated the most attractive smile as the one with no gingival recession, and the least attractive were those with 2.0 and 2.5 mm of recession. Finally, according to the laypersons, the most attractive smiles were the ones with no gingival recession and those with 0.5 mm of recession, and the least attractive were those with gingival recession of 1.5, 2.0, and 2.5 mm (Table 2, ANOVA and Tukey’s *post-hoc* test).

**Table 2:** Means and standard deviations of smile attractiveness scores of the oblique view, according to the three groups of evaluators.

Gingival Recessions	Orthodontists (O)			Periodontists (P)			Laypersons (L)			O x P x L
	Mean	SD	Result*	Mean	SD	Result*	Mean	SD	Result*	**
0.0 mm	74.82	23.08	A	80.84	20.52	A	75.42	21.08	A	O = P = L
0.5 mm	63.48	23.58	B	66.19	22.21	B	65.61	19.79	A,B	O = P = L
1.0 mm	45.93	20.68	B	48.37	17.87	C	55.59	18.50	B,C	(O = P), (P = L), O < L
1.5 mm	40.58	22.64	B,C	44.72	17.76	C,D	47.63	22.51	C,D	O = P = L
2.0 mm	36.27	20.46	B,C	35.51	20.53	D,E	43,82	22.20	C,D	O = P = L
2.5 mm	29.13	22.48	C	32.41	21.99	E	38.53	23.92	D	O = P = L

* Letters arranged in order according to smile attractiveness (ANOVA and Tukey’s post-hoc test): same capital letters in the column do not differ statistically.

**Comparison among orthodontists, periodontists, and laypersons (ANOVA and Tukey’s post-hoc test).

Comparing the opinions of the three groups of evaluators, in the frontal view, they showed statistical differences in most situations, with the orthodontists and periodontists being more critical than laypersons. In contrast, when assessing the smiles in the oblique view, no statistically significant difference was found between these groups of evaluators (Tables 1 and 2, ANOVA and Tukey’s *post-hoc* test).

When comparing the smiles in the frontal and oblique views, the evaluators showed different behavior. For the periodontists, no statistically significant difference was found between the scores assigned to the smiles in the frontal and oblique views (Table 3, Student *t* test). According to the orthodontists and laypersons, statistically significant difference was found, respectively, in few situations and in most situations, with evaluators assigning lower scores to the smiles in the oblique view (Table 4 and 5, Student *t* test).

**Table 3:** Means and standard deviations of smile attractiveness scores of the frontal and oblique views according to the periodontists.

Periodontists (P)					
Gingival recessions	Frontal view		Oblique view		P*
	Mean	SD	Mean	SD	
0.0 mm	82.96	20.40	80.84	20.52	0.42
0.5 mm	62.84	19.02	66.19	22.21	0.32
1.0 mm	53.86	21.23	48.37	17.87	0.1
1.5 mm	39.74	18.36	44.72	17.76	0.07
2.0 mm	32.19	20.16	35.51	20.53	0.12
2.5 mm	31.92	21.19	32.41	21.99	0.81

* Student t-test for the comparison between the frontal and oblique smile images according to the periodontists.

**Table 4:** Means and standard deviations of smile attractiveness scores of the frontal and oblique views according to the laypersons.

Laypersons (L)					
Gingival recessions	Frontal view		Oblique view		P*
	Mean	SD	Mean	SD	
0.0 mm	78.88	17.59	75.42	21.08	0.22
0.5 mm	70	17.75	65.61	19.79	0.28
1.0 mm	68,93	18.80	55.59	18.50	0.0044**
1.5 mm	56.48	20.42	47.63	22.51	0.0079**
2.0 mm	51.48	24.57	43,82	22.20	0.02**
2.5 mm	49.23	25.40	38.53	23.92	0.0025 **

* Dependent t-test for the comparison between the frontal and oblique smile images according to the laypersons.

** Statistically significant difference (p< 0.001).

**Table 5:** Means and standard deviations of smile attractiveness scores of the frontal and oblique views according to the orthodontists.

Orthodontists (O)					
Gingival recessions	Frontal view		Oblique view		P*
	Mean	SD	Mean	SD	
0.0 mm	73.81	26.18	74.82	23.08	0.71
0.5 mm	59.73	25.94	63.48	23.58	0.21
1.0 mm	52.01	22.81	45.93	20.68	0.01**
1.5 mm	40.80	19.71	40.58	22.64	0.91
2.0 mm	32.81	21.85	36.27	20.46	0.11
2.5 mm	34.61	20.51	29.13	22.48	0.01**

*Dependent t-test for the comparison between the frontal and oblique smile images according to the orthodontists.

** Statistically significant difference (p< 0.001).

## DISCUSSION

The results of the present study demonstrated that the presence of unilateral gingival recession on maxillary canine significantly influences the perception of smile esthetics. Orthodontists and periodontists detected all levels of recession (0.5 to 2.5 mm) in both views. Recessions of 1.5 mm and 1.0 mm were detected by laypersons in frontal and oblique views, respectively. Although studies with the same methodology were not found in the literature, Musskopf et al.^14^ also evaluated the role of gingival recessions on smile esthetics perception. They evaluated six manipulated smiles in the frontal view, with different recessions in the esthetic zone, and found that a 2-mm unilateral canine gingival recession was rated as unattractive. The present results corroborate this finding, since for all groups of evaluators and in both smile views, a 2.0-mm unilateral canine gingival recession was also rated as unattractive. Although this information is clinically important, the authors^14^ did not evaluate recessions smaller than 2.0 mm, as addressed in the present study.

Another interesting aspect is that the study of Musskopf et al.^14^ used frontal smile images. However, as suggested by Berto et al.^28^ and used by Machado et al.^27^, the oblique assessment should be considered, because in many personal relationships an oblique view of the face is commonly seen. Therefore, the present study included an oblique view in the evaluation of the smiles. It was observed that there was greater tolerance towards the presence of gingival recession in the frontal view, whereas there was greater rigor when analyzing the oblique view. In other words, evaluators (except periodontists) were less critical in their evaluations in the frontal than in the oblique evaluations. It can be hypothesized that since the number of variables in a frontal view is greater and the structures evaluated are bilateral, the possibility of a single gingival recession negatively influencing the smile is smaller. In contrast, in an oblique view, since the canine occupies a central position in the smile, gingival recession probably has a stronger negative influence on the smile esthetic perception. 

Another interesting study that also addressed the influence of unilateral alterations on maxillary canines gingival margins was the one from Correa et al.^13^ They observed that, in a frontal view of the smile, asymmetries between canine gingival margins from 1.5 to 2.0 mm were detected by laypersons. Despite the fact that these findings were similar to those from the present study and the study of Musskopf et al.^14^, it is necessary to discuss the methodology used in the above studies. Correa et al.^13^ digitally altered smiles, creating unilateral gingival asymmetries by reducing the size of clinical crown of one maxillary canine. In other words, asymmetries were created by the coronal displacement of gingival margin on one side, causing a progressive decrease in canine crown length. In the present study and in the study of Musskopf et al.^14^, the gingival margins were apically displaced, leading to an increase in canine crown length and root surface exposure. It can be stated that although the methods used by those studies were different, results were similar. In summary, it seems that laypersons’ threshold for unilateral canine gingival asymmetries is between 1.5 and 2.0 mm, either by moving gingival margin apically or coronally.

A positive aspect of the present study was that the apical displacement of the gingival margin was created in a patient who had natural gingival recessions, favoring the anatomical shape, and thus making it more realistic. Furthermore, the patient also had 3.0 mm of posterior gingival display, which made it easier for the evaluators to visualize gingival recessions in all manipulated images (0 to 2.5 mm). If the patient did not have posterior gingival display, it is obvious that these recessions would not be visible during smile evaluation. This aspect is of clinical great importance, because the results from Musskopf et al.^14^ and the present study cannot be generalized for all patients - they are aimed at patients who have minimum of 1.5 - 2.0 mm of gingival exposure at smile. 

The need for treatment of gingival recession due to esthetic concerns is evident in the present study. Gingival recession is no longer treated only to protect dental tissue or because of dentin hypersensitivity, non-carious cervical lesions or root caries.^22^ ^-^ ^24^ From a clinical point of view, it can be said that these results can influence decisions about whether to propose corrective treatment for gingival recession and also choose the best option among several types of treatment reported in the literature, such as: connective tissue graft, guided tissue regeneration, orthodontic extrusion followed by cosmetic restoration, etc.^22^ ^-^ ^24^ If the presence of unilateral gingival recession on canines of up to 1.0 - 1.5 mm, from an esthetic point of view, may not influence the laypersons’ perception, such treatments for purely esthetic reasons may seem to be an exaggerated concern.^12^ ^,^ ^27^ Obviously, since periodontists are specialists, they can detect any level of recession, however clinical decisions cannot be based only on the opinions of specialists, and patients’ chief complaints should be taken into consideration during treatment planning.

In the present study, an interesting aspect evaluated was the scores assigned by different groups in the frontal and oblique views. Based on the scores given by orthodontists, periodontists, and laypersons to the smiles in the frontal view, in most situations, orthodontists and periodontists were more rigorous than laypersons, which corroborate several articles.^4^ ^,^ ^6^ ^,^ ^7^ ^,^ ^11^ ^,^ ^13^ ^,^ ^17^ ^,^ ^19^ ^,^ ^20^ ^,^ ^27^ In general, when comparing smiles in the oblique view, no significant statistical difference among the three groups was found. Interestingly, when assessing the most attractive smiles (symmetrical and 0.5 mm recession), in both views, no statistical difference was found, which was in agreement with some previous studies.^6^ ^,^ ^9^ ^,^ ^13^ ^,^ ^19^ ^,^ ^20^ ^,^ ^27^ It can be hypothesized that an ideal smile can easily be recognized as attractive by any group of evaluators.^19^ In contrast, when small deviations occur, they start to show differences in their judgments. Another interesting finding is that periodontists did not show any statistical difference when comparing the smiles in the frontal and oblique views. It can be inferred that since they are specialists in gingival esthetics, when any recession is present the type of view do not influence their smile perception.

It is important to point out that this study used manipulated images, which make them artificial. In addition, only one smile image was used in two different views with specific groups of evaluators. Thus, as stated by Kokich et al.,^17^ since the results and conclusions are based on averages and due to the subjectivity of smile esthetics, it is difficult to customize this information to a patient. Therefore, the present study support their suggestion to discuss these results with patients who are seeking for dental esthetic treatments, especially those with maxillary unilateral canine gingival recessions.

## CONCLUSION

Based on the results obtained, it was concluded that:

» Orthodontists and periodontists perceived all levels (0.5 - 2.5 mm) of unilateral canine gingival recessions, whereas according to the laypersons, gingival recession > 1.5 mm in the frontal smile and > 1.0 mm in the oblique smile were considered unesthetic.
» Based on the scores given by orthodontists, periodontists and laypersons to the smiles in the frontal view, in most situations, orthodontists and periodontists were more rigorous than laypersons. When comparing smiles in the oblique view, no significant statistical difference was found among the three groups.
» When comparing the scores from the frontal and oblique views, no statistical difference was found in the periodontists group. Laypersons and orthodontists behavior did not follow a specific trend.
» It is important to highlight that the results of this study are aimed at patients who have minimum of 1.5 - 2.0 mm of gingival exposure at smile. On the contrary, if patients do not have gingival display at smile, any type of gingival recession will not be visualized by others.
